# β2SP/TET2 complex regulates gene 5hmC modification after cerebral ischemia

**DOI:** 10.1111/jcmm.17060

**Published:** 2021-11-19

**Authors:** Xiaohua Ma, Meng Zhang, Rui Yan, Hainan Wu, Bo Yang, Zhigang Miao

**Affiliations:** ^1^ Institute of Neuroscience Soochow University Suzhou City China; ^2^ Department of Anesthesiology The Second Affiliated Hospital of Soochow University Suzhou City China; ^3^ College of Forestry Nanjing Forestry University Nanjing City China

**Keywords:** 5hmC, ischemic stroke, OGD, TET2, β2SP

## Abstract

βII spectrin (β2SP) is encoded by Sptbn1 and is involved in the regulation of various cell functions. β2SP contributes to the formation of the myelin sheath, which may be related to the mechanism of neuropathy caused by demyelination. As one of the main features of cerebral ischemia, demyelination plays a key role in the mechanism of cerebral ischemia injury. Here, we showed that β2SP levels were increased, and this molecule interacted with TET2 after ischemic injury. Furthermore, we found that the level of TET2 was decreased in the nucleus when β2SP was knocked out after oxygen and glucose deprivation (OGD), and the level of 5hmC was reduced in the OGD+β2SP KO group. In contrast, the expression of β2SP did not change in TET2 KO mice. In addition, the 5hmC sequencing results revealed that β2SP can affect the level of 5hmC, the differentially hydroxymethylated region (DhMR) mainly related with the Calcium signalling pathway, cGMP‐PKG signalling pathway, Wnt signalling pathway and Hippo signalling pathway. In summary, our results suggest that β2SP could regulate the gene 5hmC by interacted with TET2 and will become a potential therapeutic target for ischemic stroke.

## 1 INTRODUCTION

Spectrin is the main component of the cytoskeleton and includes α‐spectrin (α1, α2) and β‐spectrin (β1‐5). βII spectrin (β2SP) is encoded by Sptbn1, and it consists of two tandem calponin homology domains (CH1 and CH2), and both contains one actin‐binding domain (ABD) in the amino‐terminus.^1^ β2SP is involved in the regulation of various cell functions, such as proliferation, blood vessel formation and immune response.^2^ More recently, β2SP has been linked to multiple signalling pathways, including cell cycle regulation,^3^ apoptosis,^4^ DNA repair,^5^ Wnt signalling,^6^ Hippo signalling,^7^ Notch signalling,^8^ β‐catenin signalling^9^ and TGF‐β signalling.^10^ β2SP also has a critical role in the nervous system. Incorrect positioning or deletion of β2SP destroys the cytoskeleton of neuronal dendrites, reduces axons^11^, ^12^ and affects the formation of the myelin sheath, which may be related to the mechanism of neuropathy caused by demyelination.^13^ As one of the main features of cerebral ischemia, demyelination plays a key role in the mechanism of cerebral ischemia‐related injury. Therefore, we believe that β2SP is involved in the mechanism of ischemic injury. There are several reports on the role of α‐spectrin in ischemic injury,^14^, ^15^, ^16^ but studies of β2SP are lacking.

Ten eleven translocation (TET) protein families were discovered by Rao and colleagues in 2009^17^ and include TET1, TET2 and TET3. TET1 is expressed in differentiated stem cells and the nervous system; TET2 is widely distributed, mainly in the hematopoietic system; and TET3 is mainly expressed in the colon and muscle, with little expression in brain tissue.^18^ The main functions of the three TET enzymes are to convert 5mC–5hmC. The 5hmC modification has been reported to affect the mechanism of many diseases, including stroke. The latest research shows that focal ischemia can increase the activity of the TET enzyme and catalyse the formation of 5hmC, and ascorbic acid (TET enzyme activator) treatment had a neuroprotective effect and improved the recovery of motor function after cerebral ischemia injury in mice.^19^ TET2 regulates changes in 5hmC in the promoter region of the Bdnf gene to affect the recovery of neurological function after cerebral ischemia injury, and loss of TET2 significantly increased the volume of cerebral infarction.^20^ Moreover, TET2 can regulate the expression of mitochondrial genes by catalysing the modification of mitochondrial DNA 5hmC, thereby damaging mitochondrial function upon ischemia injury.^21^ In summary, TET2 plays a critical role in the process of ischemic injury. However, the specific mechanism still needs more in‐depth research. In this article, we studied the molecular importance of the interaction between TET2 and β2SP in the brain after ischemic stroke.

## 2 MATERIALS AND METHODS

### 2.1 Mice

Male ICR mice (23–25 g) were acquired from the SLAC Company of China. Tet2 conditional knockout (CKO) mice: Tet2 gene flanked by LoxP sites (strain B6; 129S‐Tet2tm1.1Iaai/J, The Jackson Laboratory stock no. 017573) were crossed with Nestin‐cre mice (strain B6, obtained from Shanghai Model Organisms). The animal experiments in this article were acquired approve from the University Committee on Animal Care of Soochow University (NO. 202008A182), and accordance with the National Institutes of Health (NIH) animal operation guidelines.

### 2.2 Cell culture and oxygen and glucose deprivation (OGD) cell model

The PC12 cells used in this study were purchased from the American Type Culture Collection (ATCC). The cells were maintained in DMEM with 10% foetal bovine serum (HyClone) at 37°C in a 5% CO_2_ incubator. PC12 cells were incubated in sugar‐free medium and then placed in an airtight chamber (Billups Rothenberg) flushed with a mixed gas of 95% N2/5% CO_2_ for 15 min. The chamber was kept at 37°C for 1 h. Control PC12 cells were incubated with sugar‐free medium and placed in the incubator. The cells are restored to their normal culture conditions after the hypoxia is over.

### 2.3 Mouse model of middle cerebral artery occlusion (MCAO)

We refer to the previous method to prepare the MCAO models.^22^ At first, a 6–0 nylon filament was inserted into the internal carotid artery about 9–11 mm. Then, the filament was removed after 45 min, and recover reperfusion. Mice in the sham group underwent the same experimental procedures, but the nylon filament was not inserted. The mouse was placed on an electric blanket to keep the body temperature at 36.5°C–37.5°C, and monitored the brain blood flow.^23^

### 2.4 Western blot

Mouse brain tissues and PC12 cells were solubilized and desaturated. We previously described our analysis of protein expression.^20^ Anti‐β2SP rabbit antibody (1:1000, Abcam, ab72239, RRID:AB_1270902), anti‐TET2 rabbit antibody (1:1000, Abcam, ab124297, RRID:AB_2722695), anti‐Histone 3 rabbit antibody (1:2000, Immunoway, YM3038), anti‐β‐tubulin mouse antibody (1:8000, Sigma, sab4200715, RRID:AB_2827403) and anti‐β‐actin mouse antibody (1:2000, HUAAN, M1210‐2) were used.

### 2.5 Immunofluorescence staining

Mouse brain was cut into 15‐μm thick sections after perfusion and dehydrated; the PC12 cells were fixated with paraformaldehyde. We incubated the primary antibody (anti‐β2SP mouse antibody, 1:200, Santa Cruz, sc‐515592; anti‐TET2 rabbit antibody, 1:500, Abcam, ab124297, RRID:AB_2722695) with the sections after incubation with blocking buffer. Then, we incubated secondary antibodies (Jackson ImmunoResearch Laboratories) at 37°C for 1 h. The photographs were acquired with fluorescence microscope.

### 2.6 Dot blot

Genomic DNA was extracted from brain tissues and cells for dot blot analysis by the phenol chloroform method. We accordance the previous method of dot blot to test 5hmC levels.^24^ Anti‐5hmC rabbit antibody (1:10000, Active Motif, 39769, RRID:AB_10013602) was used.

### 2.7 Methylene blue staining

After dot blot, the membrane was incubated with methylene blue (0.02% in 0.3 M sodium acetate, Sigma‐Aldrich Company) for 10 min.^25^ Photograph was acquired after washing, and the results were analysed with Alpha Ease Image Analysis Software.

### 2.8 Coimmunoprecipitation (Co‐IP)

Brain tissues and PC12 cells were dissolved in lysis buffer. The samples were centrifuged to collect the supernatant. The supernatant was incubated with anti‐TET2 overnight at 4°C. Then, the supernatant was precipitated with protein A/G‐agarose beads (Santa Cruz, sc‐2003, RRID:AB_10201400) for 4 h at 4°C. Beads were collected, and protein samples were obtained for western blotting.

### 2.9 β2SP sgRNA transfection

sgRNAs target β2SP was designed with CRISPR Guide RNA design tool Benchling, then cloned into lenti‐CRISPRv2(addgene#52961). The Guide RNAs are listed in Table 1. PC12 cells were transfected with 500 ng plasmid and 1.5 μL of Lipofectamine 3000 reagent (Thermo Fisher) in DMEM (HyClone). We used western blotting and gDNA sequence to detect the effectiveness of Cas9 sgRNA after 48 h of transfection. Then, a β2SP KO clone was chosen to perform other experiments.

**TABLE 1:** The sequence of sgRNA for β2SP

Primer	Sequence
β2SPsg 2 R	aaacTGCCAAAACCCACCAAGGGC
β2SPsg 2 F	CACCGCCCTTGGTGGGTTTTGGCA
β2SP sg 8 R	aaacTTGGTGGGTTTTGGCAGGGTC
β2SP sg 8 F	CACCGACCCTGCCAAAACCCACCAA
β2SP sg 16 R	aaacTGGTGGGTTTTGGCAGGGTC
β2SP sg 16 F	CACCGACCCTGCCAAAACCCACCA
β2SP sg 17 R	aaacGTACAGCGACCTGCGGGACGC
β2SP sg 17 F	CACCGCGTCCCGCAGGTCGCTGTAC
β2SP sg 20 F	CACCGTGAATCCGCATCCGGCCCT
β2SP sg 20 R	aaacAGGGCCGGATGCGGATTCAC

### 2.10 5hmC‐specific capture and analysis

We follow the previous method to mark 5hmC,^26^ coupled with high‐throughput sequencing. The sequencing data were processed using previous method.^27^ Briefly, Bowtie 2^28^ and Samtools^29^ softwares were used to deal the FASTQ sequence data, and mapping the data with Mus musculus reference genome (mm10). There were not allowed to appear more than two mismatches in the first 25 bp, and retained one non‐repetitive genome. We counted the unique and non‐repetitive reads of enriched sample genomic DNA in the 100, 1,000 and 10,000 bp and non‐enriched input genomic DNA in whole genome bins, and normalized these data to the total number of repeated reads. This study used ngsplot^30^ software to analyse the coverage of each sample reads in the genomes, and the Model‐based Analysis of ChIP‐Seq (MACS2)^31^ was used to determine the true regions or “peaks” of 5hmC‐enriched.

### 2.11 GO and KEGG analyses

GO analyses were to deal with the Database for Annotation, Visualization and Integrated Discovery (DAVID) analysis tools. KEGG analyses were to deal with Kyoto Encyclopedia of Genes and Genomes (KEGG) software.

### 2.12 Statistics

All data are expressed as the mean ±SEM. GraphPad Prism 6.0 (GraphPad Software, Inc.) was used for statistical analysis. Student's *t*‐test and one‐way analysis were used to determine the differences in the groups. *p* < 0.05 was considered statistically significant.

## 3 RESULTS

### 3.1 β2SP is increased after ischemic stroke

**FIGURE 1:**
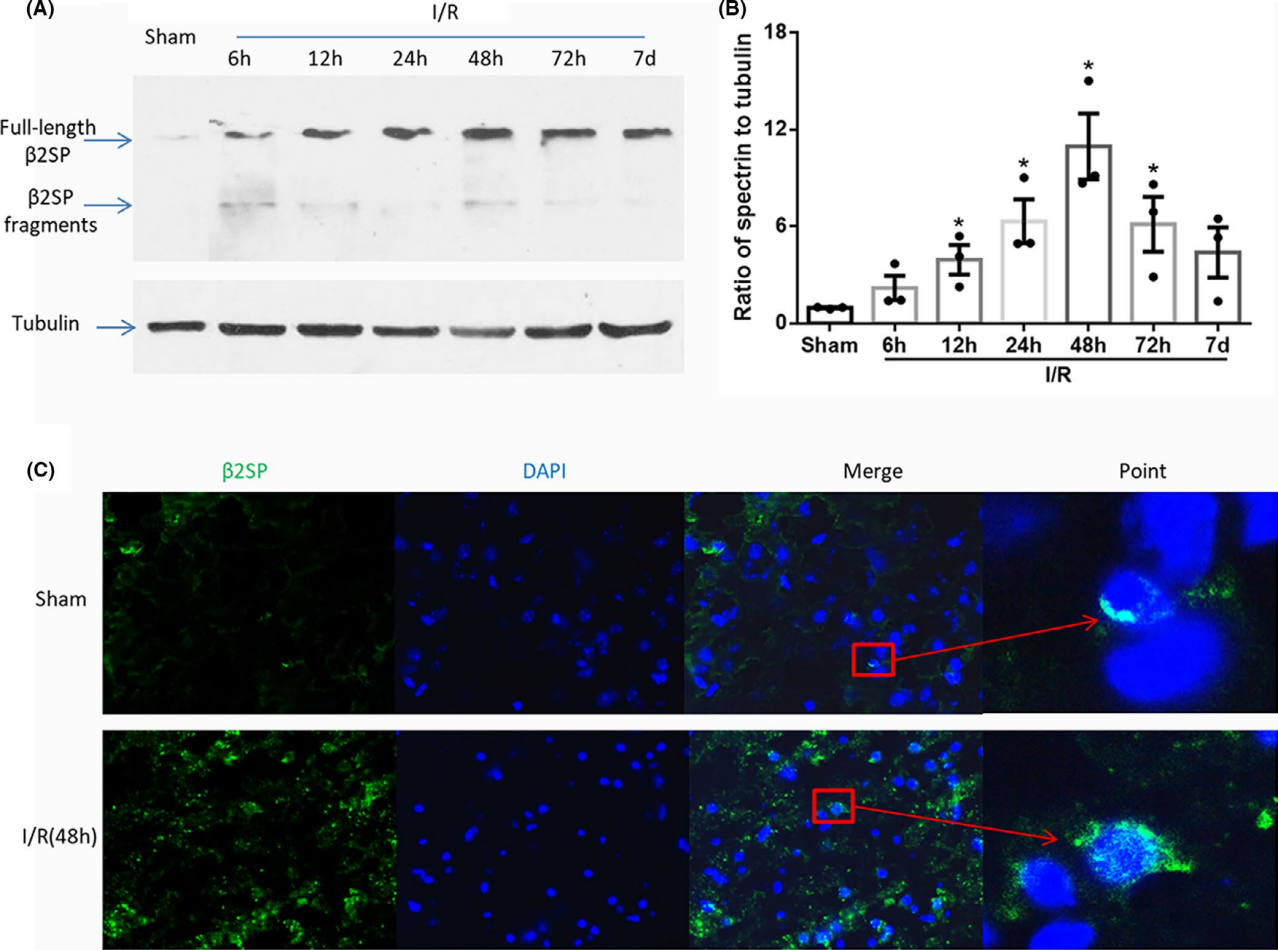
The level of β2SP was increased after cerebral ischemia/reperfusion injury. (A) shows representative western blot staining of β2SP, and statistical analysis of the relative intensity is shown in (B). The bottom panel shows the immunofluorescence staining of β2SP at 48 h after I/R injury (C). *p < 0.05

At present, the level of β2SP is still unknown after cerebral ischemic injury. To explore this, we used the MCAO model and western blot method to detect the β2SP levels in ischemic tissues. The different time points (6, 12, 24, 48 h and 7 days) were set, and the results showed that the β2SP levels were increased and reached peak at 48 h (Figure 1A), which was confirmed by quantitative analysis (*p* < 0.05, Figure 1B). This result was consistent with our previous findings on 5hmC.^20^ Moreover, fluorescence staining demonstrated an increase in β2SP at 48 h, and β2SP may have transferring to nucleus (Figure 1C). These results indicated that the expression and distribution of β2SP were changed after ischemic injury.

### 3.2 β2SP is transferred to the nucleus and increases 5hmC levels after OGD

**FIGURE 2:**
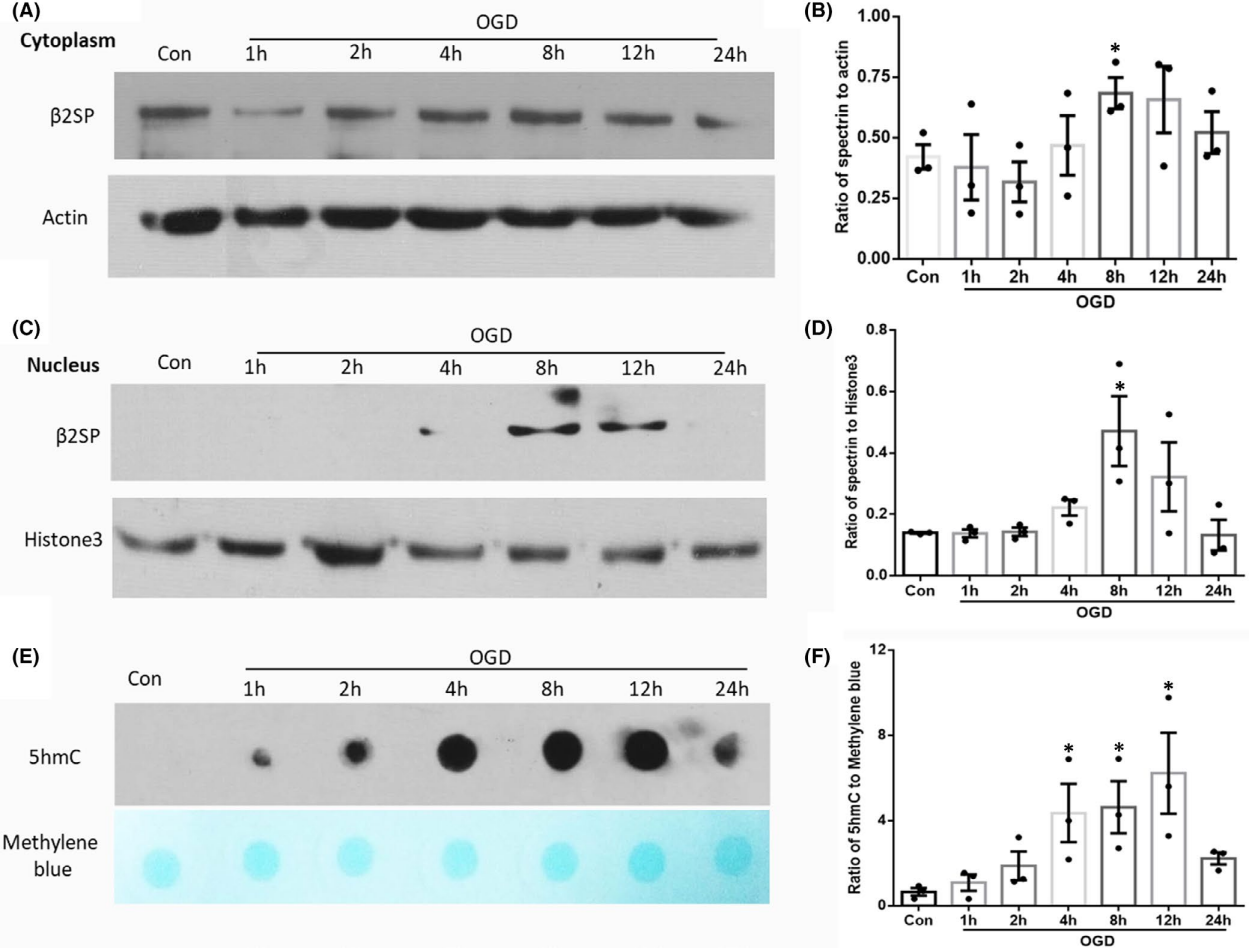
The expression of β2SP was specifically upregulated in the nucleus after OGD. (A and C) The protein levels of β2SP in the cytoplasm and nucleus at different time points (1, 2, 4, 8, 12 and 24 h) after OGD. Actin and Histone 3 were used as a control. The statistical analyses of the relative intensity are shown in (B and D). (E) The 5hmC levels were detected by dot blots at different time points after OGD, and the statistical analysis of the relative intensity is shown in F, *p < 0.05

Previous articles have shown that the adaptor protein β2SP plays an essential role in the nucleus to drive TGFβ‐mediated tumour suppression,^32^ and the nuclear accumulation of β2SP was decreased in cirrhotic liver tissue.^33^ These results indicated that β2SP can enter the nucleus and play a key role in cell signalling. To further study the mechanism by which β2SP enters the nucleus after ischemic injury, we used an OGD cell model. First, we detected the expression of β2SP in the cytoplasm and nucleus at different time points (1, 2, 4, 8, 12 and 24 h) after OGD. β2SP levels were increased at 8 h in the cytoplasm (Figure 2A), which was confirmed by quantitative analysis (*p* < 0.05, Figure 2B). In the nucleus, β2SP expression appeared at 4 h, reached its peak at 8 h and disappeared at 24 h (Figure 2C); this finding was confirmed by quantitative analysis (*p* < 0.05, Figure 2D). To explore whether β2SP affects 5hmC modification after entering the nucleus, we detected the levels of 5hmC at different time points (1, 2, 4, 8, 12 and 24 h) after OGD. The results indicated that the levels of 5hmC were increased and peaked at 12 h (Figure 2E,F), which was consistent with the entry of β2SP in the nucleus. These results suggest that β2SP enters the nucleus and may affect 5hmC levels after OGD.

### 3.3 β2SP interacts with TET2 after OGD

**FIGURE 3:**
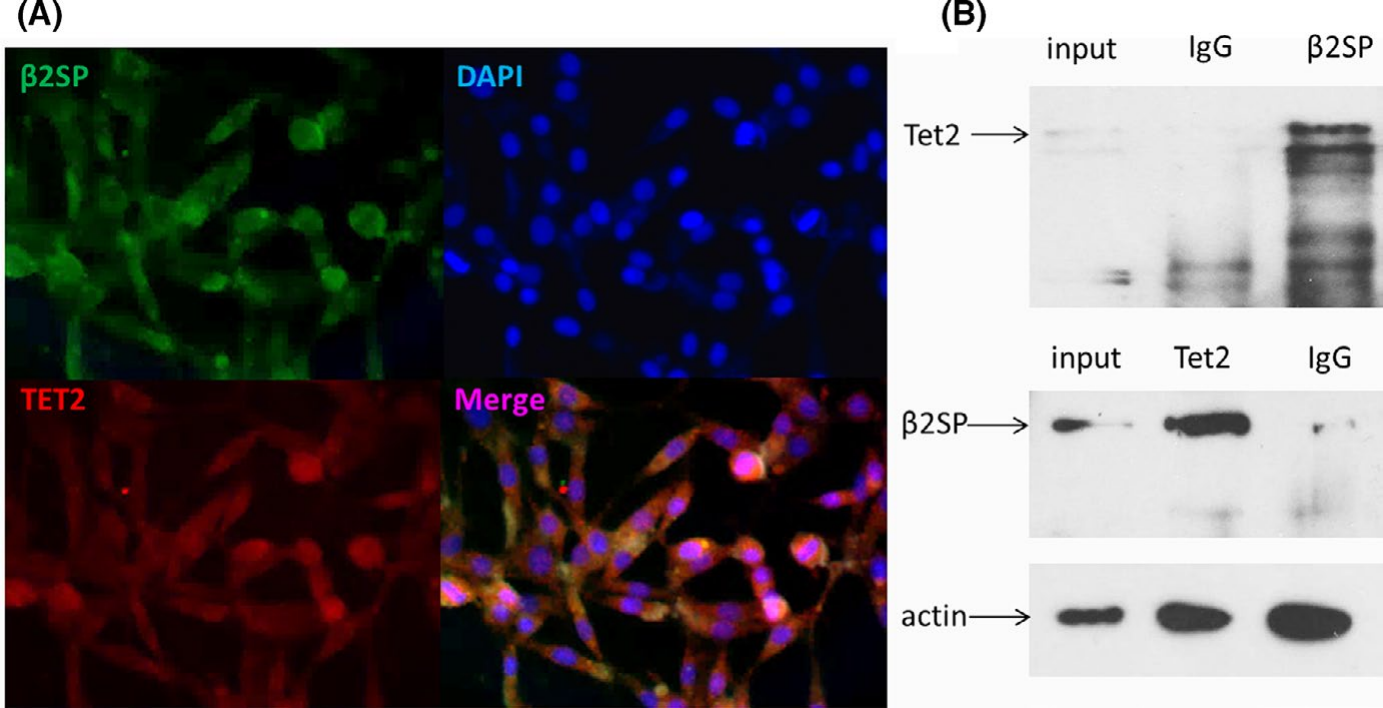
βII spectrin interacted with TET2 after ischemic injury. (A) Immunofluorescence staining of β2SP and TET2 8 h after OGD. (B) The interaction between β2SP and TET2 was detected by Co‐IP

Our previous results suggested that the increase of 5hmC was correlated with TET2 after ischemic injury.^20^, ^21^ Therefore, we hypothesized that TET2 interacts with β2SP after ischemic injury. To explore this, we first examined the distribution of β2SP and TET2 in the OGD model by fluorescence staining. At different time points (2, 8 and 24 h) after OGD, β2SP and TET2 co‐localized inside or outside the nucleus, and they showed the same trend at different time points (Figure 3A). Furthermore, Co‐IP demonstrated that TET2 interact with β2SP (Figure 3B).

### 3.4 β2SP knockout decreases TET2 and 5hmC levels after OGD

**FIGURE 4:**
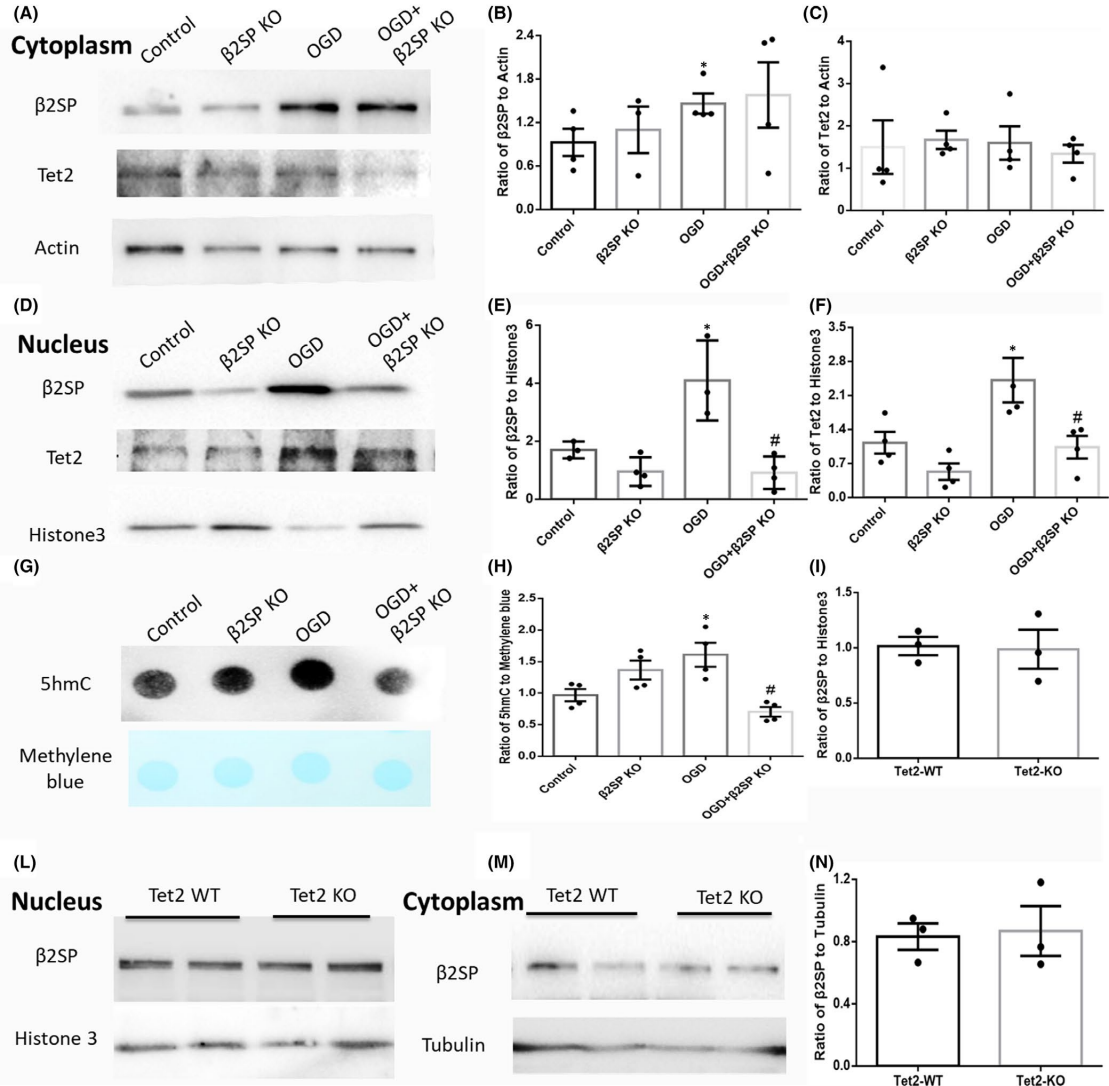
βII spectrin knockout decreased the TET2 and 5hmC levels after OGD. (A and D) The protein levels of β2SP and TET2 in the cytoplasm and nucleus when β2SP was knocked out after OGD. Actin and Histone 3 were used as a control. The statistical analysis of the relative intensity is shown in (B, C, E and F). (G) The level of 5hmC was detected when β2SP was knocked out after OGD, and statistical analysis of the relative intensity is shown in H. (L and M) The protein levels of β2SP in the cytoplasm and nucleus in the mice after OGD. Actin and Histone 3 were used as controls, and the statistical analysis of the relative intensity is shown in I and N * was compared with the control group, # was compared with the OGD group, ^*^ *p* and ^#^ *p* < 0.05

From the above experimental results, we know that β2SP increases significantly in the nucleus and interacts with TET2 after ischemic injury. How TET2 change when β2SP is deleted? To explore this topic, we detected changes in the TET2 and 5hmC levels when β2SP was knocked out after OGD. First, we designed 5 sgRNA (Table 1) for the Cas9 knockout experiment in PC12 cells. Then, β2SP knockout cells were obtained through screening (Figure S1), and we used these cells for the next experiment. OGD modelling was performed, and the cells were collected to extract cytoplasmic, nuclear protein and genomic DNA for western blot and dot blot experiments after 8 h of incubation. The western blot results indicated that the levels of TET2 were decreased in the nucleus and not changed in the cytoplasm when β2SP was knocked out after OGD (Figure 4A–F). As shown by the dot blot results, the levels of 5hmC were reduced in the OGD+β2SP KO group (Figure 4G,H). These results confirmed that β2SP can affect TET2 translocation to the nucleus to convert 5mC to 5hmC. But the levels of β2SP did not change in the nucleus or cytoplasm in the Tet2 WT and KO mice after MCAO (Figure 4I–N). These results indicated that TET2 was not necessary for β2SP nuclear transfer and that its expression increased after ischemic injury.

### 3.5 The 5hmC was changed after β2SP knockout

**FIGURE 5:**
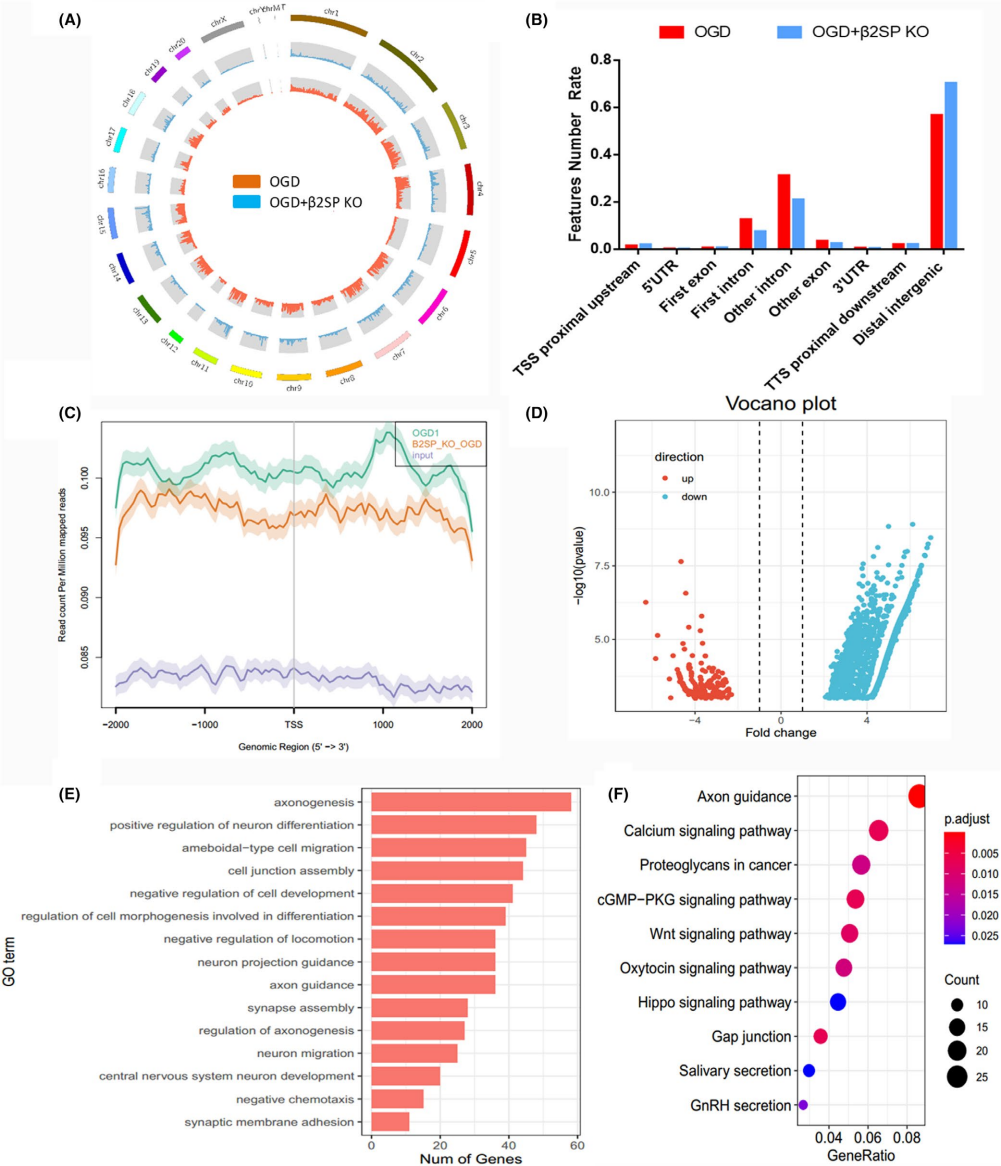
Analysis of 5hmC sequence data after β2SP KO. (A) showed the 5hmC distribution of different chromosome between the OGD and OGD+β2SP KO groups. (B) indicated the 5hmC reads distribution in different region. (C) The read count Per Million mapped reads in Genomic Region (5′ −> 3′). (D) Volcano plot of the differentially genes. Red indicated upregulated, and blue showed down‐regulated. (E) GO analysis of DhMRs. (F) KEGG analysis of DhMRs

To explore the effect of deletion of β2SP on 5hmC modification, we analyse the 5hmC distribution of genes in the β2SP KO‐PC12 cell line. The analysis results indicated that the overall 5hmC levels were lower in OGD +β2SP KO group than in OGD group (Figure 5). Figure 5A showed that the 5hmC levels in the different chromosome. The 5hmC levels significantly decreased in OGD +β2SP KO group (Figure 5A). The result of 5hmC reads distribution indicated that the levels were reduced in exon, intron and 3′UTR after β2SP KO (Figure 5B). Then, we visually analysed the read count Per Million mapped reads in Genomic Region (5′ −> 3′), and the result showed that the level was decreased in OGD +β2SP KO group (Figure 5C). These results showed that the 5hmC abundance was decreased after β2SP KO.

To further study the different genes with significant changes in 5hmC distribution after β2SP KO, we used the 5hmC sequence data to analyse the differentially hydroxymethylated regions (DhMRs). The results showed that there are 2,234 genes were down‐regulated and 1,275 genes were upregulated after β2SP KO (Figure 5D). Then, we used DAVID analysis tools to perform Gene Ontology (GO) analysis, and used KEGG software to analysis KEGG pathway. KEGG analysis found that the DhMRs were highly related with cell connection signalling pathway, such as Calcium signalling pathway, cGMP‐PKG signalling pathway, Wnt signalling pathway and Hippo signalling pathway (Figure 5F). GO analysis showed that the DhMRs were related with axonogenesis, positive regulation of neuron differentiation, ameboidal‐type cell migration and cell junction assembly (Figure 5E).

## 4 DISCUSSION

Spectrin family proteins have major domains: CH domains include the CH1 and CH2 domains; coiled‐coil repeats represent a long and short helix per repeat; pairs of EF‐hands have a number indicating the number of pairs; and a WW domain and ZZ domain are also present.^34^ β2SP can interact with many proteins through these domains. This molecule binds to Scribble (a tumour suppressor protein) by CH1,^35^ interacts with CCI through the long C‐terminal variant,^36^ interacts with Smad3 via the N‐terminal fragment,^37^ binds to ankyrin with the C‐terminus^38^ and so on. β2SP exerts its specific functions through these interactions, including regulation of the cell cycle, apoptosis and transcription. Our results showed that β2SP interacts with TET2 to affect the levels of 5hmC after ischemic injury, but the domain to which they bind has not been identified, and more work is needed to explore the specific domain. Some studies found that full‐length αII‐spectrin protein was decreased and αII‐spectrin breakdown products (αII‐SBDPs) were significantly increased in brain tissue after ischemic injury^39^, ^40^; these SBDPs play a key role in ischemic injury.^41^, ^42^ Currently, there is no related report of β2SP in ischemic stroke, and only one article showed that full β2SP(260 kDa) is breakdown to 110 kDa, 108 kDa, 85 kDa and 80 kDa fragments in the rat brain (hippocampus and cortex) after traumatic brain injury.^43^ In our studies, full‐length β2SP was increased significantly, but β2SP breakdown products (βsBDPs) were not significantly changed (Figure 1A) after I/R injury.

The roles and functions of 5hmC have been studied in many diseases since 2010, when the TET protein family was identified. Different TET proteins play various functions in different diseases, but TET2 has been reported to play a more important role in ischemic injury. Our previous study showed that the levels of 5hmC were increased, and TET2 protein deletion could increase the Infarct volume after ischemic brain injury.^20^ The mtDNA 5hmC abundance is increased after ischemic brain injury and may be associated with the expression of mitochondrial genes and cellular ATP levels.^21^ In addition, TET2 was reported to be dispensable for kidney development and function under baseline conditions while protecting against renal IR injury, possibly by repressing the inflammatory response,^44^ and TET2 can affect the levels of cell death‐related genes by regulating the 5hmC levels after spinal cord injury.^45^ However, there are other reports that overall 5hmC abundance in the cortex was decreased significantly, and the reduced expression of Tet1 and Tet2 enzymes might be responsible for this change after hypoxic‐ischemic injury^46^; moreover, Kahlilia C et al.^19^ thought that TET3 regulated 5hmC to provide endogenous neuroprotection against cerebral ischemia. Although 5hmC plays a key role in cerebral ischemic injury, the specific mechanism is not yet clear. Our results confirmed that β2SP can regulate the levels of TET2 in the nucleus and affect the levels of 5hmC after ischemic injury.

βII spectrin as an adaptor protein formed by the Smad3/Smad4 complex in the process of TGF‐β signal transduction,^47^ and participates in the regulation of multiple signal pathways. Wnt signalling was activated after SPTBN1 lose,^48^ βIV‐spectrin can mediate function and excitability through to bind and recruit Ca^2+^/calmodulin kinase II^49^ and Spectrin can regulate the Hippo signalling pathway.^7^ Our results showed that the 5hmC modifications of 2,234 genes have a significant decrease after β2SP KO in OGD cell model. KEGG analysis result showed that the DhMRs were highly related with Calcium signalling pathway, cGMP‐PKG signalling pathway, Wnt signalling pathway and Hippo signalling pathway. These results were basically the same as previously reported in the literature. These results suggest that β2SP may regulate the 5hmC modify of genes to affect the corresponding signalling pathways through bind with TET2.

In summary, we show that the levels of β2SP were increased after ischemic injury, and these increases were mainly in the nucleus. Immunofluorescence staining and Co‐IP analyses suggested an interaction between β2SP and TET2. β2SP KO reduced the levels of TET2, but TET2 KO did not affect the expression of β2SP after ischemic injury, highlighting the key role of β2SP in ischemic injury. Our data suggest that β2SP could regulate the gene 5hmC by interacting with TET2 and will become a potential therapeutic target for ischemic stroke.

## CONFLICT OF INTEREST

We have no conflicts of interest to declare.

## AUTHOR CONTRIBUTION

**Xiaohua Ma:** Supervision (equal); Writing‐original draft (lead). **Meng Zhang:** Data curation (equal). **Rui Yan:** Formal analysis (equal). **Hainan Wu:** Data curation (equal). **Bo Yang:** Project administration (equal). **Zhigang Miao:** Writing‐review & editing (equal).

## Supporting information

Fig S1
