# Supplementary figures and images for: β2SP/TET2 complex regulates gene 5hmC modification after cerebral ischemia
Source: J Cell Mol Med. 2021 Nov 19;25(24):11300–9. doi: 10.1111/jcmm.17060 (PMC8650033; doi:10.1111/jcmm.17060)

**A**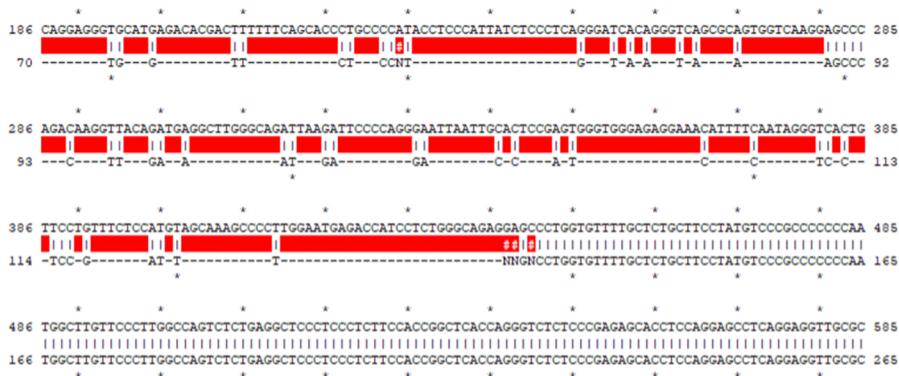**B**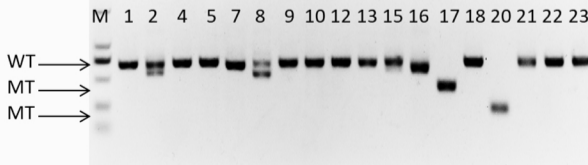**C**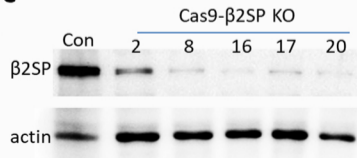

Supplement: Supplementary file 1 — Fig S1 [file JCMM-25-11300-s001.pdf]
